# Exploring women's experiences with persistent pain and pain management following breast cancer treatment: A qualitative study

**DOI:** 10.3389/fpain.2023.1095377

**Published:** 2023-02-13

**Authors:** Michelle D. Smith, Joanne Manning, Mandy Nielsen, Sandra C. Hayes, Melanie L. Plinsinga, Michel W. Coppieters

**Affiliations:** ^1^ School of Health and Rehabilitation Sciences, Physiotherapy, The University of Queensland, Brisbane, QLD, Australia; ^2^ Acquired Brain Injury Transitional Rehabilitation Service, Division of Rehabilitation, Princess Alexandra Hospital, Brisbane, QLD, Australia; ^3^ Menzies Health Institute Queensland, Griffith University, Brisbane and Gold Coast, QLD, Australia; ^4^ Faculty of Behavioural and Movement Sciences, Amsterdam Movement Sciences, Vrije Universiteit Amsterdam, Amsterdam, Netherlands

**Keywords:** breast cancer, treatment side effects, neuropathic pain, management, cancer pain

## Abstract

This qualitative study aimed to explore experiences of women with persistent pain following breast cancer treatment, including their perceptions about the cause of their pain, how they manage their pain and their interactions with healthcare providers related to their pain during and following breast cancer treatment. Fourteen women who experienced pain for more than 3 months following breast cancer treatment were recruited from the general breast cancer survivorship community. Focus groups and in-depth, semi-structured interviews were conducted by one interviewer, audio-recorded, and transcribed verbatim. Transcripts were coded and analysed using Framework Analysis. Three main descriptive themes emerged from the interview transcripts: (1) characteristics of pain, (2) interactions with healthcare providers and (3) pain management. Women had various types and degrees of persistent pain, all of which they believed were related to breast cancer treatment. Most felt like they were not given enough information pre- or post-treatment and believed their experience and ability to cope with pain would have been better if they were given accurate information and advice about (the possibility of) experiencing persistent pain. Pain management strategies ranged from trial and error approaches, to pharmacotherapy, and to ‘just coping with the pain”. These findings highlight the importance of the provision of empathetic supportive care before, during and after cancer treatment that can facilitate access to relevant information, multidisciplinary care teams (including allied health professionals) and consumer support.

## 1. Introduction

Breast cancer is the most common cancer in women, with over 1.3 million cases diagnosed each year worldwide (1), including more than 20,000 Australians (2). The overall five year survival rate in developed countries exceeds 90% (3, 4). However, the majority (90%) of women report at least one persistent adverse effect six months post-breast cancer treatment, and at six years post-breast cancer, more than 60% of cancer survivors (survivorship defined as time from cancer diagnosis until the end of life (5)) report one or more adverse effect amenable to rehabilitative intervention (6, 7).

Persistent pain, defined as pain lasting more than three months (8), represents a common breast cancer treatment sequalae, reported by up to 60% of women following breast cancer treatment (9–11). It is associated with decreased quality of life, function, and higher healthcare costs (12–14). Persistent pain has also been identified as a cause of discontinuing or adjusting various specific breast cancer treatments like changes to aromatase inhibitors, and therefore potentially influences risk of recurrence, disease progression and mortality (15, 16).

Previous qualitative studies suggest that women are both unprepared to experience persistent pain and are dissatisfied with pain management information provided by their healthcare providers (17, 18). Despite increased access to health information from a variety of sources, including the internet (19, 20), healthcare providers remain an essential source of information, guidance and reassurance to patients (17). The majority of the available literature comes however from the United States and European countries, which have vastly different healthcare systems, cultures, and languages. To guide implementation of findings and clinical practice in a way that benefits Australian women with breast cancer, it is therefore important to also understand experiences of persistent pain in the Australian healthcare system, and specifically the relationships between women and their healthcare providers in terms of information exchange, level of support received at different stages of breast cancer treatment and the extent to which their breast cancer survivorship needs are being met. Therefore, the aim of this qualitative study was to further explore experiences of breast cancer survivors with persistent pain, including their perceptions about the cause of their pain, how they manage their pain and their interactions with healthcare providers regarding their pain during and following breast cancer treatment.

## 2. Material and methods

### 2.1. Design

This was an original, stand-alone, exploratory qualitative study with a descriptive inductive design in a sample of breast cancer patients with persistent pain. Convenience sampling was used to recruit people from the Brisbane, Gold Coast and Sunshine Coast areas in Australia. We conducted semi-structured interviews designed to explore women's experiences with persistent pain, including their perceptions about the cause of their pain, how they manage their pain and their interactions with healthcare providers regarding their pain. Participants were interviewed on a single occasion. As such, our methodology was grounded in constructivism which considers reality to be affected by people's experiences and thoughts. All interviews were guided by the semi-structed interview guide which allowed for the development of new ideas as they were introduced (21).

### 2.2. Participants

Participants were eligible for inclusion if they were female, over 18 years of age, proficient in English, diagnosed with primary breast cancer, received or were receiving treatment (surgery/radiation/chemotherapy/hormone therapy) for breast cancer, and reported pain for longer than three months. Women with known metastases or breast cancer as a secondary tumour were excluded. Ethical approval was obtained from The University of Queensland Medical Research Ethics Committee. All participants provided written informed consent prior to participating in the study.

### 2.3. Procedure

The research team consisted of researchers and clinicians with experience and expertise in persistent pain, pain management, cancer survivorship and qualitative study methods. Participants were invited to participate in the study through emailing members of the Breast Cancer Network Australia Review and Survey Group, and *via* advertisement on social media platforms, newsletters and posters. Eighteen women expressed interest to participate in the study, of whom four were excluded because they did not meet the inclusion criteria (*n* = 2) or did not have time to participate (*n* = 2). We were able to interview 14 participants who were all given the choice to participate in either a focus group, or in individual semi-structured interviews. Four participants opted for the focus group and ten participants for the semi-structured interviews.

All interviews were conducted by a female investigator (JM) who had a background in Physiotherapy and creative arts and was trained to conduct the interviews by an experienced qualitative researcher. A second investigator attended the interviews to take field notes about interactions between interviewer and interviewee and the physical environment. The second investigator was either a social worker with expertise in qualitative research and chronic pain (MN) or a physiotherapist trained in qualitative methodology (MS). There were no prior relationships between the interviewers and any of the interviewees and participants were not financially compensated for their participation. The *a priori* developed semi-structured interview guide was followed. The focus group and interviews were audio-recorded and transcribed verbatim by the researcher who conducted the interviews. Recruitment, data collection and analysis proceeded concurrently until data saturation was reached (i.e., when no new consistent themes were emerging) (22). Participants did not comment on transcripts or initial findings.

### 2.4. Other measures

Participants also completed a short questionnaire to obtain information about demographics, history of breast cancer diagnosis and treatment, pain, and pain interference with function. Location of pain was identified on a body chart. Worst and average intensity of pain experienced in the past month were rated on 11-point numerical rating scales, anchored with the words “no pain” at 0 and “worst possible pain” at 10. Additional numerical rating scales anchored with the words “not at all” at 0 and “completely” at 10 were used to rate how much pain interfered with function, participation in recreational and workplace activities, and overall enjoyment of life.

### 2.5. Data analysis

De-identified transcripts were imported into the qualitative analysis program QSR NVivo 10 for Mac (QSR International Pty Ltd, Doncaster, Australia). To identify, describe and interpret recurrent patterns around persistent pain, pain management and experiences with healthcare professionals, the data were analysed inductively using the Framework Analysis. Framework Analysis involved five stages – familiarisation to gain an overview of the data, initial theme identification, indexing and sorting data into themes, reviewing, and summarizing (23). To improve trustworthiness, two investigators independently developed preliminary theme lists. The two lists were then discussed between the two researchers to ensure themes were grounded in the data. A theme was considered a final theme when it captured experiences relating to pain, pain management or experiences with healthcare professionals, and when it was noted across a number of transcripts. The study adheres to the consolidated criteria for reporting qualitative research (COREQ) checklist to confirm rigour (24).

## 3. Results

### 3.1. Participant characteristics

The fourteen included women were all English speakers residing in Australia, had a mean (standard deviation (SD); range) age of 56 (7; 42–67) years and were on average 4.5 (2.4) years post-diagnosis. The majority of women received radiotherapy or chemotherapy in addition to surgery, as well as hormone therapy (Table 1). The median number of pain sites was 7 (range 1–12 sites), and women reported a wide range of pain locations, the most common being the anterior breast, lateral breast/chest/axilla, and the foot/ankle (Table 2). Data for pain intensity and interference with function are presented in Table 3.

**Table 1:** Participant characteristics (*n* = 13) presented as mean (standard deviation) or number (percentage).

Characteristic	
Age (years)	55.8 (7)
Body mass index (kg/m^2^)	27.9 (5.2)
Years since diagnosis	4.5 (2.4)
**Treatment received**	
Lumpectomy	9 (69%)
Unilateral mastectomy	5 (38.5%)
Bilateral mastectomy	2 (15.4%)
Axillary/sentinel node removal	13 (100%)
Breast reconstruction	1 (7.7%)
Radiation therapy	10 (76.9%)
Chemotherapy	8 (61.5%)
Hormone therapy	11 (84.6%)

**Table 2:** Number (percentage) of women who report pain following breast cancer treatment in the different body areas (*n* = 13).

Pain location	Ipsilateral	Bilateral	Contralateral
Anterior breast	6 (46)	2 (15)	0 (0)
Lateral breast/chest/axilla	7 (53)	2 (15)	0 (0)
Shoulder	4 (31)	3 (23)	0 (0)
Upper arm	1 (8)	0 (0)	0 (0)
Wrist/hand	1 (8)	4 (31)	1 (8)
Upper back	1 (8)	0 (0)	0 (0)
Abdomen	0 (0)	2 (15)	0 (0)
Hip	0 (0)	4 (31)	0 (0)
Knee	0 (0)	3 (23)	1 (8)
Foot/ankle	0 (0)	6 (46)	1 (8)

**Table 3:** Pain characteristics measured with the numeric rating scale (NRS), ranging from 0 to 10 (higher scores indicate worse pain or function) (*n* = 13).

	Mean (SD)
**Pain intensity NRS**	
Worst pain in last month	5.7 (2.5)
Average pain in last month	4.1 (2.0)
**Pain interference with function NRS**	
General activity	4.0 (3.3)
Lifting ability	6.4 (3.2)
Relationships with other people	3.1 (3.0)
Enjoyment of life	3.8 (3.3)

Abbreviations: SD, standard deviation.

One 90 min focus group was conducted with four participants. Telephone (*n* = 9) and in-person (*n* = 1) interviews of 10–50 min in length were conducted for women who preferred this data collection method.

### 3.2. Qualitative analysis

Framework Analysis identified three key descriptive themes related to pain and pain management: (1) characteristics of pain, (2) interactions with healthcare providers, and (3) pain management strategies. Representative quotes from participants are used to illustrate the findings throughout the text and in Supplementary Table S1. Numbers are used to distinguish participants (e.g., P1, P2 … P14).

#### 3.2.1. Theme 1: characteristics of pain

*Distribution and description of pain.* Women described their pain as “*excruciating”, “aching”, “throbbing”, “cramping”, “pins and needles”, “sharp”, “numb and shooting”, “tender”* and “*stabbing”*. One participant who underwent a double mastectomy reported phantom breast pain and persistent “crushing pain”. Five women reported joint pain, one describing it as if someone had “*hit [her] in the kneecap with a hammer”* [P12].

Women generally attributed their pain to some form of breast cancer treatment after surgery, like chemotherapy or radiotherapy, for example: “*I probably didn’t have a lot of pain, but probably – not after the initial surgery; I was really good”* … “*well, the initial radiation you have the pain from, like any of the burning sensation. And then the ongoing pain is ongoing on the side where I had the radiotherapy”* [P8]. Women attributed pain to a particular treatment due to the area of pain, what they read or heard from others, and timing of onset, for example, “*I didn’t have much pain with the operation - the main pain came when I had the Arimidex, when I was put on that…”* [P5]. However, due to the combination of treatments, some women were uncertain what caused their pain, and others questioned whether ageing was a factor. Due to the unexpected onset and uncertainty about the cause, participants identified “*an element of anxiety”* [P1] associated with their pain and concern that it signaled a return or metastasis of the cancer. One participant spoke of the difficulty in managing the balance “*between hypochondria and just being sensible about things”* [P4].

#### 3.2.2. Theme 2: interactions with healthcare providers

Participants saw a range of healthcare providers during their cancer treatment which included general medical practitioners, surgeons, oncologists, nurses, physiotherapists and osteopaths.

Participants were not always satisfied with the information they received from their healthcare providers prior to commencing their breast cancer treatment. Despite specifically asking about consequences of treatment, participants indicated they were not told about the possibility of experiencing pain. Women were surprised to experience persistent pain: “*I’ve had shocking side effects in many ways from the treatment… and I was not warned about any of this”* [P2]. In contrast, other participants indicate they were told about the possibility of experiencing some pain. Prior to receiving radiotherapy one participant was told she would have pain for a few years, but she was not informed it could last longer than this. Another woman was told she may “*get a few aches and pains”* [P12] as a side effect of hormonal therapy, but she did not feel this accurately reflected the “*extreme pain”* [P12] she experienced. Some women also acknowledged that due to being “*stuck”* [P14] on the diagnosis at the time, they were unsure if they had been told about the possibility of experiencing pain post-treatment. Overall, women believed they should be better informed prior to treatment that they might experience persistent pain as a side effect. Women felt being armed with this information in advance would have helped them manage their pain and prepare for the future, for example “*but I don’t know if they let you know that it's like an ongoing thing”* [P8].

Women consistently indicated that when they approached their healthcare providers about the pain during and following their breast cancer treatment they were constantly dismissed and their healthcare providers appear uninterested: “*When I told the oncologist after three weeks he said ‘No, it is too early for you to be feeling that sort of pain’”* [P10], and “*When I did say that I had pain, they kept saying ‘You shouldn’t have pain’, which I didn’t really appreciate”* [P5]. Some participants said their healthcare providers denied the pain was related to treatment and subsequently ignored it. Sometimes healthcare providers disagreed on what was causing the pain: “*The chemo doctor said it's not his chemo. The rheumatologist disagrees with the chemo doctor”* [P12]. Other participants were told the pain was “*just normal”* [P20] or “*just nerve damage”* [P6], and no management was offered, with participants quoting their healthcare providers saying: “*You shouldn’t still be here and be happy that you are”* [P2], and “*Get on with your life”* [P6].

Overall, most women felt their healthcare providers were not listening to their concerns. Women voiced a sense of despair and abandonment that following completion of breast cancer treatment they were left alone to deal with the consequences, for example “*So you get to the end of what's a very intense six, nine months and then bang, it's nothing”* [P1]. Some participants indicated their healthcare providers suggested pain management options. Many women were told to use pharmacology, including paracetamol, non-steroidal anti-inflammatory drugs, sleeping tablets or a combination of drugs, to manage their pain. Some women were “*eventually”* referred to other healthcare providers, one woman was referred to a pain clinic, and ceasing treatment was suggested for another woman. Commonly, women stopped turning to their healthcare providers for help and sought out other sources such as the internet to try to understand and manage their pain: “*I found a website with women from all over the world complaining about exactly the same pain as I had so I felt, oh… I’m not going [crazy]”* [P5].

#### 3.2.3. Theme 3: pain management

Participants heard about management options from a variety of sources including the Cancer Council, literature from hospitals, other breast cancer survivors, and more commonly “*Dr Google”* [P12]. Most women adopted a trial and error approach to pain management, using a wide range of strategies, including: “*massage, the TENS [Transcutaneous electrical nerve stimulation] machine, ice packs, heat packs, [prescription narcotics], anti-depressants…”* [P5], as well as resistance training, weights, hydrotherapy, Tai Chi, stretching, meditation and dietary changes. Participants most commonly used and recommended exercise (walking, weights, Pilates, yoga, stretching, kayaking, exercise classes) as a pain management strategy, and many women reported using massage and heat (i.e., hot showers/baths and hydrotherapy). After trying numerous pain management options with limited success, many participants felt there was nothing that could be done to alleviate their pain: “*I think I’ve just given up, and… I’ve just got to deal with [it] and just live with it.”* [P6] Some women concluded that pain was just an inevitable outcome of being treated for breast cancer. Feeling that nothing could be done to help them, many participants kept the pain to themselves and coped as best they could.

Although some women were told to use pharmacology for their pain, participants acknowledged a hesitance to take medication to help with pain relief: “*I’ve got all these drugs going in me now…I don’t think I want a sleeping pill”* [P9]. One participant was unable to function on the analgesics prescribed, and therefore opted to not take them. Although some women did not want to use pharmacology, many would take medication if the pain was “*really bad”* [P7], or if nothing else worked. Women used pharmacology for pain relief with varying degrees of success.

## 4. Discussion

Identified themes suggest that participants in our study experienced pain in a range of body locations, including the breast, axilla, upper and lower limbs, all of which participants believed was related to breast cancer treatment. Novel contributions of this study include women feeling that there is a lack of adequate and consistent information on the relationship between cancer and persistent pain pre- and post-treatment, feeling a lack of health professional recognition and empathy regarding persistent pain, and a lack of support in accessing relevant strategies in the Australian healthcare system. Specifically, it appeared that most women felt as if they were not given enough information pre- or post-treatment and believed their experience and ability to cope with pain would have been better if they were given accurate information and advice about (the possibility of) experiencing persistent pain. Pain management strategies ranged widely, and included trial and error approaches, pharmaceutical management approaches and to “just coping with the pain”.

One of the main concerns identified by women was the lack of information from healthcare providers about pain and their surprise at experiencing *persistent* pain following breast cancer treatment. These findings support previous qualitative findings in which women reported being surprised and uncertain of whether pain was a normal part of treatment (25), and poorly informed at diagnosis and throughout treatment about the possibility of experiencing pain (18). In the current study, most women believed their experience and ability to cope with the pain could have been improved if they were educated about the possibility of experiencing pain in advance. Although women were surprised to experience persistent pain after breast cancer treatment, they acknowledged they may have been told about it at the time of diagnosis but not absorbed the information due to focusing on the diagnosis and medical procedures to follow. This suggests multiple formats of patient education, such as written and verbal information, are required to enhance information retention. There is evidence that cancer survivors who receive appropriate patient-centred information report better health-related quality of life and lower levels of anxiety and depression (26) and improvements in treatment-related side effects, including pain (27).

As well as lack of information, women reported lack of health professional recognition and empathy regarding persistent pain. Indeed, a cross-sectional study in over 100 breast cancer patients in an urban medical centre in the United States identified that discussions about treatment-related side effects are rarely initiated by healthcare providers (28). Participants in our study report a lack of acknowledgement, and even denial, from healthcare providers about their pain being related to breast cancer treatment. Many women simply wanted acknowledgement that the pain they experienced may be related to their breast cancer treatment and some explanation about the cause of the pain. This finding is supported by a recent review that included four qualitative studies conducted in breast cancer survivors in Sweden, France and Norway with acute and chronic pain which reported that some women felt dismissed after mentioning their persistent pain to their healthcare provider (18). Interestingly, these experiences are not only found in various healthcare systems and cultures around the globe, but also mirror that of the broader population of people with non-cancer persistent pain. Studies consistently report lack of understanding and acknowledgement by healthcare providers of the persistent pain a patient experiences (29, 30).

Women felt a lack of support in accessing relevant treatment options in the Australian healthcare system. Educating patients on pain neurophysiology and neurobiology has been shown to have a positive effect on pain and disability (31, 32). With up to 60% of women following breast cancer treatment experiencing pain (9–11), this breast cancer treatment-related side effect lends itself for early education and prospective monitoring. However, in light of this not occurring, women turned to other sources, such as the internet and other breast cancer survivors, to help them try to manage their pain. Through trial and error, some women found a specific strategy that helped them cope with their pain; whereas, others could not do anything about it, and learned to live with it. Interestingly, participants in our study identified a reluctance to use pharmacology to try to relieve their pain, despite this being the most common management strategy provided by their healthcare providers. This is supported by Fenlon et al. (33) who found women post-breast cancer take less pain-relieving medication than women without breast cancer. Given the hesitance to take pharmaceuticals and mixed effectiveness of pain management strategies (34–37), it is important for healthcare providers to provide a range of pain management options for women who have been treated for breast cancer. Evidence suggests that best-practice pain management involves coordinated interdisciplinary care (38). Data suggest that this is not occurring in women with persistent pain following breast cancer treatment, and these women are not likely receiving optimal pain management.

This study has some limitations that should be considered. The study design may have skewed the sample toward women who are comfortable discussing their pain experiences and toward women who have worse experiences with pain. This limits applicability to other contexts, but findings can still provide considerable insights for patients, clinicians and researchers about persistent pain (management) in breast cancer survivors. Our study design allowed participants to choose between participating in a focus group or in an interview. These different approaches may have impacted our findings; however, the same interview guide was used and there was no observation that responses differed between the interview or focus group participants. This study was conducted in Southeast Queensland and findings may not be transferable to settings in rural areas or other countries and their cultures. The depth of the data resulting from the interviews is likely impacted on by the nature of the participants, and any prompting statements made by the interviewers. Not having participants comment on the interpretation and study findings prior to publication is unlikely to have influenced the main findings, trends and themes identified in our study. However, benefits of having participants comment on the interpretation and study findings may include refining of language around themes and subthemes. All participants were women and breast cancer survivors, and therefore findings may have limited transferability to men or other people with other cancer types or treatments. Most interviews were conducted *via* telephone, and therefore we were not always able to note non-verbal communication.

The findings of this study demonstrate that women experience persistent pain in a range of body locations, all of which they believe was a consequence of breast cancer treatment. Participants felt they were given inadequate and inconsistent information, support and management advice from their healthcare providers, resulting trial and error pain management approaches, ranging from pharmacological approaches through to ‘just live with it’. These findings support what has been found in other healthcare systems. Given the consistency in findings and increasing survival rates of women with breast cancer, future research needs to develop accessible and effective pain management interventions that guide clinical practice in a way that benefits women.

## Data Availability

The raw data supporting the conclusions of this article will be made available by the authors, without undue reservation.
