# Supplementary material for: Exploring women's experiences with persistent pain and pain management following breast cancer treatment: A qualitative study
Source: Front Pain Res (Lausanne). 2023 Feb 13;4:1095377. doi: 10.3389/fpain.2023.1095377 (PMC9968918; doi:10.3389/fpain.2023.1095377)
Supplement: Supplementary file 1 [file Table1.docx]

Supplementary Material

# Supplementary Table 1. Representative quotes from participants in each theme and subtheme (n=14).

| Theme | Participant number |
| --- | --- |
| *Theme 1. Characteristics of pain* |  |
| Distribution and description of pain  “…even just having a sheet on my toes or something can really hurt…and I just can’t sleep.”  “…mainly joint pain, ankles, knees, wrists and my hips.” | 5  7 |
| Attributed cause of pain  “And this was mainly after or during, but certainly after the radiotherapy”  “…it was the aromatase inhibitors. I went on them just before I started radiation and then it was three weeks and I was complaining to the oncologist that I was in a lot of pain’  “I guess my biggest issue apart from the actual site that I had the mastectomy was following the chemo, and Herceptin. I ended up with chronic pain in my feet…”  “Is this the…metastasizing that takes off now…?”  “I can never really pinpoint what thing it’s from, whether it’s from the chemotherapy which just about did me in, or whether it’s just arthritis, or whether it’s one of the side effects of Arimidex, which – joint and muscle ache is one of the major and most commonly experienced side effects. So it’s frustrating I can never say what it is, and therefore how I should approach it.” | 11  10  6  4  3 |
| *Theme 2. Interactions with healthcare providers* |  |
| Information provided pre-treatment  “I think the question you asked before about whether we were told about the pain after treatment is quite interesting, because I think I would have been a little bit more prepared if there was that simple statement.”  “I don’t believe I was given the information to make an informed choice.” | 3  2 |
| Pain management options post-treatment  “From my experience, there’s no-one really listening to those consequences. It’s, oh well this is the treatment, if you want to survive have the treatment and deal with the side effects.”  “There doesn’t seem to be any – any recognition or acknowledgment that it’s not as simple as you die or you get better. I think there are probably a lot more women in the same limbo sort of area where we are not dying but our lives are completely – totally changed.” | 9  5 |
| *Theme 3. Pain Management* |  |
| Approach to pain management  “I’ve done resistance training, weights. I have tried the pool... Tai Chi… stretches…meditation a couple of times a week. Eat copious amount of turmeric…and pretty much run my diet around whatever’s going on with my body.”  “I have to go kayaking to get relief…and then that gives me two weeks”  “I started yoga thinking that might help with the shoulder and the arm, and it actually made it worse…so I had to stop” | 12  1  9 |
| Perspectives about pharmacology  “I was a person who didn’t even take an aspirin until I had cancer. So the whole experience of having to have these drugs put in my body in the first place was a big one for me…as soon as I could reduce whatever I could reduce that’s what I did…I’ve got Panadeine Forte and a few other bits and pieces that I can take if things get too much…But generally naturally things are better...I just don’t like taking drugs” | 2 |
| “Just coping” with the pain  “Well, there’s nothing anyone can do about it”  “Get on with life. That's what I do. Get on with life and listen to other people whinging about minor things and in my mind think, you think you've got something to worry about, but I don't say anything. What else can you do?”  “I think I’ve just given up, and I just think, well, I’ve just got to deal with and just live with it.” | 9  2  6 |
