# Supplementary material for: Exploring women's experiences with persistent pain and pain management following breast cancer treatment: A qualitative study
Source: Front Pain Res (Lausanne). 2023 Feb 13;4:1095377. doi: 10.3389/fpain.2023.1095377 (PMC9968918; doi:10.3389/fpain.2023.1095377)
Supplement: Supplementary file 2 [file Table2.docx]

**Appendix 1.**

**Semi-structured Interview/Focus Group Guide**

Opening:

Introductory:

Transition:

Key Questions:

Ending Questions:

**1.** Tell us your first name and briefly about what prompted you to come along today.

**2.** Can you tell me about your experience with pain following breast cancer treatment?

- Did a healthcare provider inform you that you might experience pain?
- Why do you think you have this pain?
- What do you think is the cause of your pain?

**3.** In what ways does your pain impact your life?

**4.** What have you tried to manage your pain?

- How did you hear about this?
- Have you ceased a treatment due to the pain you felt it caused?

**5.** What was the effect of this type of management on your pain?

**6.** Describe what effect this type of management has had on your daily life

- Work/housework/social life

**7.** Has this type of management had any effect on what breast cancer treatments you have received or are receiving?

**8.** Is there anything else that you’d like to say on the subject, that you didn’t get a chance to say?
